# Blood lipids and lipoproteins in relation to incidence and mortality risks for CVD and cancer in the prospective EPIC–Heidelberg cohort

**DOI:** 10.1186/s12916-017-0976-4

**Published:** 2017-12-19

**Authors:** Verena Andrea Katzke, Disorn Sookthai, Theron Johnson, Tilman Kühn, Rudolf Kaaks

**Affiliations:** 1 Division of Cancer Epidemiology, German Cancer Research Center (DKFZ), Im Neuenheimer Feld 581, 69120 Heidelberg, Germany; 2 Translational Lung Research Center Heidelberg, Member of the German Center for Lung Research (DZL), Heidelberg, Germany

**Keywords:** Lipoprotein, Lipids, Cholesterol, Cancer, Mortality, EPIC–Heidelberg

## Abstract

**Background:**

Circulating concentrations of lipid biomarkers are associated with risk of cardiovascular diseases (CVD). The evidence for a relationship with cancer risk, however, is not entirely consistent. This study aims to assess the relationships of total cholesterol (TC), high-density lipoprotein cholesterol (HDL-C), triglycerides (TG), apolipoprotein (a) (apo(a)), apoB-100, and lipoprotein(a) (Lp(a)) with risk of common cancer forms and total cancer mortality in comparison to incidence and mortality of CVD.

**Methods:**

We selected a case-cohort sample out of the prospective EPIC–Heidelberg study, including a random subcohort (*n* = 2739), and cases of cancer (*n* = 1632), cancer mortality (*n* = 761), CVD (*n* = 1070), and CVD mortality (*n* = 381). Concentrations of lipid biomarkers were measured in pre-diagnostic blood samples. Hazard ratios (HR) and 95% confidence intervals (CI) were estimated using Prentice-weighted Cox regression models.

**Results:**

High levels of circulating apoB-100 and TG were inversely associated and high HDL-C levels were positively associated with breast cancer risk (highest vs. lowest quartile (Q4 vs. Q1), HR_apoB_ 0.71, 95% CI 0.52–0.98; HR_TG_ 0.65, 0.46–0.92; and HR_HDL_ 1.39, 1.01–1.93). Higher levels of Lp(a) were associated with an increase in prostate cancer risk (Q4 vs. Q1, HR_Lp(a)_ 1.43, 1.02–2.03) and high levels of apo(a) were associated with a decrease in lung cancer risk (Q4 vs. Q1, HR_apo(a)_ 0.52, 0.30–0.91). High TC, HDL-C, apo(a), and Lp(a) levels were associated with a reduction in total cancer mortality (Q4 vs. Q1, HR_TC_ 0.71, 0.54–0.94; HR_HDL_ 0.67, 0.50–0.91; HR_apo(a)_ 0.71, 0.54–0.93; and HR_Lp(a)_ 0.74, 0.57–0.98). All lipid biomarkers were associated with risk of myocardial infarction, whereby TC, apoB-100, TG, and Lp(a) were positively and HLD-C and apo(a) inversely associated with risk. Only high levels of TG were associated with an increased risk of stroke. None of the lipids were associated with risk of colorectal cancer and with risk of CVD mortality after multivariable adjustments.

**Conclusions:**

This prospective study demonstrates inverse associations of lipid biomarkers with cancer incidence and mortality, with the exception of positive associations of HDL-C and Lp(a) with breast and prostate cancer risk, respectively. Thus, the observed cancer risk pattern clearly differs from the CVD risk pattern.

**Electronic supplementary material:**

The online version of this article (doi:10.1186/s12916-017-0976-4) contains supplementary material, which is available to authorized users.

## Background

Circulating concentrations of lipid biomarkers are consistently associated with cardiovascular disease (CVD) events and thus are considered major indicators of metabolic health. In particular, high levels of low-density lipoprotein cholesterol (LDL-C) have been consistently associated with up to 1.7-fold increases in risk of CVD in observational studies [1]. Other lipid parameters, such as high-density lipoprotein cholesterol (HDL-C), triglycerides (TGs), or lipoprotein (a) (Lp(a)), have also been increasingly investigated in relation to risk of atherosclerotic CVD or coronary heart disease [1–4].

Lipids and lipoproteins in blood, including LDL-C and HDL-C, are responsible for cholesterol metabolism in humans. HDL-C is a small and dense lipoprotein, containing proteins and lipids, which removes fat molecules and cholesterol from cells and transports them back to the liver for excretion or re-utilization. Apo(a) is the major protein component of HDL-C in plasma and enables efflux of fat molecules by accepting fats from within cells. In contrast, apoB-100 is the primary apolipoprotein of chylomicrons, very low-density lipoprotein, intermediate-density lipoprotein, and LDL particles, and as such is responsible for transport of fat molecules to all peripheral cells. Another major component of chylomicrons and very low-density lipoprotein are TGs, which play an important role as transporters of dietary fat and as energy sources. In contrast to these lipid biomarkers, the metabolism of Lp(a) is largely unknown. Lp(a) is commonly described as an LDL-like lipoprotein particle consisting of one apo(a) molecule covalently bound to apoB-100 [5]. While high HDL-C and apo(a) levels have been associated with decreased relative risks for CVD in the range of 0.7 to 0.8, up to 1.5-fold increased risks were generally observed for high levels of total cholesterol (TC), appB-100, TG, and Lp(a) [1, 3, 6]. Although uncertainty still exists with regard to HDL’s causal role [7], a pattern of high LDL-C, TG, and Lp(a) levels, and possibly of low HDL-C levels, is widely used for CVD risk stratification.

With regard to cancer incidence, the associations of lipid biomarkers, and particularly of apolipoproteins (apo) and Lp(a), have been investigated much less thoroughly than for CVD, and results from different prospective studies performed thus far are not entirely consistent. Elevated blood concentrations of TC and TG and lower concentrations of HDL-C and apo(a) were associated with an increased risk of overall cancer in the most recent meta-analysis based on 28 epidemiologic studies [8], with relative risks in the range of 1.1 to 1.4. Additionally, the latest prospective investigation within the Women’s Health Study is largely in agreement with this meta-analysis [9]. However, for common individual cancer entities such as breast, colorectal, prostate, and lung cancer, associations of risk with blood lipids are either inconsistent across prospective cohort studies or have been only scarcely investigated [9–20]. Nevertheless, several biological mechanisms have been proposed linking lipid biomarkers to overall cancer development or cancer death, including immune system disturbances, increased tumor angiogenesis, reduced tumor apoptosis, and increased tumor cell proliferation [21, 22].

Epidemiologic studies on cancer mortality are less numerous than those on CVD mortality, but low TC levels have been consistently associated with an up to 2-fold increase in overall cancer mortality in these studies [23–25]. These observations have led to uncertainty regarding the overall health benefit of cholesterol-lowering strategies at the population level. Prospective studies on the association of HDL-C, apo(a), apoB-100, and TG with overall cancer mortality are few and results conflicting [9, 26, 27]. Regarding Lp(a), low levels were found to be positively associated with overall cancer death, with a risk of 1.5 in the only prospective study conducted so far [28].

Such inconsistent results and limited investigations for some of the lipids and cancer entities make it difficult to define a common metabolic lipid profile characteristic for cancer risk similar to the one for CVD. It is also unclear whether these lipids are causally related to cancer risk or whether they are merely markers of general lifestyle determinants. In light of these uncertainties, we conducted a case-cohort study within the European Prospective Investigation into Cancer and Nutrition (EPIC)–Heidelberg cohort on the associations of lipid biomarkers with risk of common cancer forms and total cancer mortality, adjusted for multiple potential lifestyle factors. For comparison purposes, we report the associations of lipid parameters with risk of stroke, myocardial infarction (MI), and CVD death.

## Methods

### Study setting and design

We conducted a case-cohort study embedded within the EPIC–Heidelberg cohort. EPIC–Heidelberg is part of the larger European EPIC project and comprises 25,546 men and women from the local general population of Heidelberg and surroundings [29, 30]. Participants were recruited between 1994 and 1998 and were aged between 35 and 65 years. At baseline, participants provided a blood sample and information on dietary habits as well as lifestyle factors in an extensive questionnaire and in interviews; in addition, anthropometric indices were measured by trained staff. Informed consent was obtained from all participants at baseline and the Medical Ethics Committee of the Heidelberg University approved the study.

### Population and case ascertainment

In EPIC–Heidelberg, chronic disease occurrences were prospectively ascertained through active follow-up through study subjects and their next-of-kin, combined with linkages with hospitalization records, and cancer and pathology registries. Data on vital status and causes of death were collected from municipal and regional mortality registries. For the present case-cohort study, verified incident cases of breast, colorectal, prostate, and lung cancer diagnosed up to the end of 2012, verified incident cases of MI and stroke, and verified cases of death up to the end of 2009 were included. A randomly selected subcohort of approximately 10% of the entire EPIC–Heidelberg cohort participants served as the reference pool (*n* = 2739). An exclusion criterion for any subject was non-availability of a blood sample. For the current study, 627 incident cases of breast (ICD-10: C50), 554 cases of prostate (ICD-10: C61), 195 cases of lung (ICD-10: C34), and 256 cases of colorectal cancer (ICD-10: C18-20) were included. In addition, 515 incident cases of stroke (ICD-10: I60, I61, I63, I64, ischemic *n* = 406, hemorrhagic *n* = 90, unspecific *n* = 19) and 555 cases of MI (ICD-10: I21) were included. Within the full EPIC–Heidelberg cohort, 1516 participants deceased until end of follow-up (Dec 2012), of which 761 (50%) died of cancer (any type), 381 (25%) of cardiovascular events, and the remaining 374 (25%) of miscellaneous conditions. All causes of death were also coded according to the ICD-10. We chose a case-cohort design in order to economize on samples and costs and since several failure time outcomes can be analyzed with the same comparison group, as the random sample subcohort is selected independently of the outcome [31].

### Laboratory methods

Serum samples were sent on dry ice to Scandinavian Health Ltd. laboratories (Etten-Leur, Netherlands) for basic clinical chemistry measurements, including serum concentrations of TC, HDL-C, TG, apo(a), apoB-100, and Lp(a). All measurements were made using the Roche Cobas 6000 analytical system for clinical chemistry, according to the manufacturer’s protocols. A small proportion (< 10%) of the analytes were not successfully measured and therefore the number of subjects may vary depending on the applied statistical model. The Friedewald formula (LDL = TC – HDL – TG/5) was applied to calculate LDL-C values [32].

### Statistical analyses

Spearman partial correlation coefficients (r) adjusted for sex and age at recruitment were calculated in the subcohort for selected baseline variables and lipid biomarkers. For analyses on cancer risk and mortality, we applied Prentice-weighted Cox proportional hazard regression models with age as the underlying time-scale to calculate hazard ratios (HR) and 95% confidence intervals (CI). All observations in the subcohort were left-truncated at age at recruitment and right-censored at end of follow-up, death, or loss of follow-up, whichever came first. Cases were included at the time of their event, following the Prentice weighting scheme [33]. An extended version of the Schoenfeld residuals test showed no violations of the proportional hazards assumption [34]. By applying cause-specific hazard regression analyses, competing events were taken into account [35]. Lipid biomarkers entered the models continuously log 2 transformed or in quartiles, with sex-specific cut-points based upon the distribution in the cases and the lowest quartile considered as the reference category. Tests for trend of risk over lipid biomarker levels were based on the median of each quartile modelled as a quantitatively scaled statistical variable. Further analyses were conducted for dichotomized values of biomarkers, with the clinical cut-points TC ≥ 6.18, HDL ≤ 1.03, apo(a) ≤ 1.05, apoB ≥ 1.56, TG ≥ 2.26, and Lp(a) ≥ 120 used according to the National Cholesterol Education Program and European Society of Cardiology/European Atherosclerosis Society [36–38].

Prentice-weighted Cox regression models were used to estimate both crude and multivariable adjusted HR and 95% CIs for the associations between lipid biomarkers, cancer risk, risk of CVD, and mortality. The crude model includes sex and age as co-variables. Confounding factors were included in the multivariable adjusted models if they changed the HR by more than 10%, were significantly associated with either the exposure or the outcome, or were considered as important risk factors based on biological plausibility. Variables retained in adjusted models were body height, waist circumference, BMI, lifetime alcohol consumption, intake of red meat and fiber, smoking habits (never, former, current, years since quitting smoking, numbers of cigarettes smoked, age started smoking), socioeconomic status (school level), physical activity, diabetes, hypertension, and use of lipid-lowering drugs. Cox models with breast cancer as an outcome were further adjusted for oral contraceptive use, hormone replacement therapy, menopausal status, and number of full-term pregnancies. Heterogeneity by sex (colorectal and lung cancer only), current alcohol consumption at recruitment (nondrinkers vs. drinkers), menopausal status at baseline, age 55 at diagnosis and hormone replacement therapy (HRT) use at baseline (for breast cancer only), smoking status (never, former, current), and obesity was based on Q statistics, adjusted for age and, if applicable, for sex. To assess possible reverse causation bias, sensitivity analyses were conducted excluding cases diagnosed within the first 2 or 5 years of follow-up. We further excluded subjects with HRT use at baseline (*n* = 915, 34% of the female study population), those with lipid-lowering medication use (*n* = 295, 5% of the total study population), those with prevalent liver insufficiency (*n* = 79), and those with prevalent kidney insufficiency (*n* = 66) in sensitivity analyses. The last two conditions were self-reported in a questionnaire (“Has your doctor ever diagnosed chronic liver/kidney insufficiency? If yes, please indicate month and year.”). Risk associations for calculated LDL-C levels were comparable to those obtained by apoB-100 and can be found in Additional file 1. ApoB-100 is the primary apolipoprotein of LDL-C and was highly correlated with LDL-C (*r* = 0.93) in our data.

All statistical tests were two-sided and *P* values of less than 0.05 were considered statistically significant. All analyses were performed using SAS 9.4 (SAS Institute, Cary, NC, USA).

## Results

Baseline characteristics of the EPIC–Heidelberg subcohort participants are displayed in Table 1. Briefly, EPIC subcohort participants were, on average, 50 years of age at recruitment, slightly overweight, and mostly moderately inactive; 30% self-reported hypertension, while diabetes, kidney, and liver insufficiency and use of lipid-lowering drugs were less prevalent at baseline. Men in the subcohort were mostly former smokers and highly educated, while women mostly obtained a secondary school degree and prominently reported to have never smoked. Almost 40% of women were postmenopausal at baseline, of whom more than 60% were using HRT. Median follow-up time of the subcohort was 15.6 years, whereas median follow-up time of cancer incidence and mortality was the range of 7.6 to 9.4 and of CVD incidence and mortality in the range of 7.8 to 8.1 years.

**Table 1:** Characteristics of the study population (EPIC–Heidelberg, subcohort participants)

	Subcohort		
	Women	Men	Total
N	1466 (54)	1273 (46)	2739
Age at recruitment, years	49 (35–66)	53 (40–65)	51 (35–66)
BMI, kg/m^2^	25.3 ± 4.7	26.8 ± 3.6	26.0 ± 4.3
Waist circumference, cm	81 ± 12	96 ± 10	88 ± 13
Smoking (n, %)			
Never	753 (51)	435 (34)	1188 (43)
Former	406 (28)	509 (40)	915 (33)
Current	303 (21)	325 (26)	628 (23)
Physical activity (n, %)^a^			
Inactive	191 (13)	138 (11)	329 (12)
Moderately inactive	527 (36)	431 (34)	958 (35)
Moderately active	408 (28)	363 (29)	771 (29)
Active	340 (23)	341 (27)	681 (25)
Education level (n, %)			
Primary school	403 (28)	377 (29)	780 (29)
Secondary school	711 (49)	414 (33)	1125 (41)
University degree	352 (24)	482 (38)	834 (30)
Dietary variables (median, IQR)			
Alcohol consumption, mg/d	4 (2–10)	20 (11–36)	10 (3–22)
Red meat intake, g/d	19 (10–29)	33 (20–55)	25 (13–42)
Processed meat intake, g/d	34 (18–56)	56 (33–82)	44 (23–68)
Fiber intake, mg/d	18 (15–23)	20 (16–24)	19 (15–23)
Diseases and medication (n, %)^b^			
Hypertension	353 (24)	435 (34)	788 (29)
Diabetes mellitus	29 (2.0)	63 (4.9)	92 (3.4)
Kidney diseases	5 (0.3)	19 (1.5)	24 (0.9)
Liver diseases	9 (0.6)	9 (0.7)	18 (0.7)
Use of lipid-lowering drugs	47 (3.2)	72 (5.7)	119 (4.3)
Menopausal status (n, %)			
Premenopausal	653 (44.5)	–	–
Current use of pill	82 (5.6)		
Postmenopausal	552 (37.7)	–	–
Current use of HRT	366 (25.0)		
Biomarkers (median, IQR)			
Total cholesterol, mmol/L	5.80 (5.1–6.5)	5.80 (5.2–6.6)	5.80 (5.1–6.6)
HDL-C, mmol/L	1.60 (1.3–1.9)	1.20 (1.0–1.5)	1.40 (1.1–1.7)
Apo(a), μmol/L	1.79 (1.6–2.0)	1.55 (1.4–1.7)	1.67 (1.5–1.9)
ApoB-100, μmol/L	1.06 (0.9–1.2)	1.16 (1.0–1.4)	1.10 (0.9–1.3)
Triglycerides, mmol/L	1.30 (0.9–1.9)	1.90 (1.4–2.8)	1.60 (1.1–2.3)
Lp(a), nmol/L^c^	13.40 (6.1–46.4)	12.00 (5.4–42.5)	12.60 (5.8–45.4)

Values are means ± standard deviations or proportions, dietary variables and biomarker values are medians and interquartile ranges

^a^According to the Cambridge Physical Activity Index

^b^Self-reported at baseline

^c^To convert into mg/dL = nmol/L × 0.4167

*apo* apolipoprotein, *BMI* body mass index, *HDL* high-density lipoprotein, *HRT* hormone replacement therapy, *IQR* interquartile range, *Lp(a)* lipoprotein (a)

**Fig. 1:**
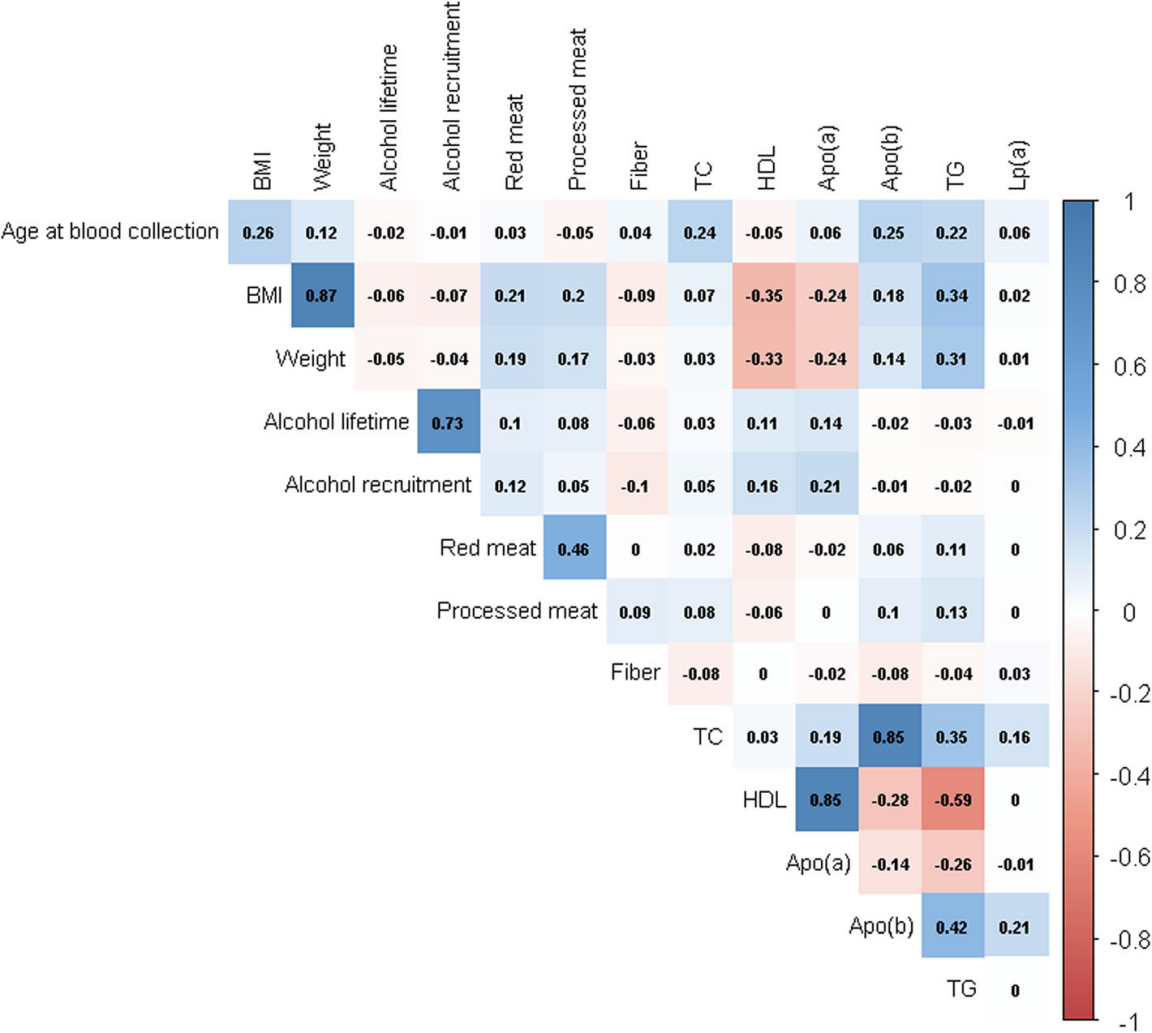
Correlation matrix of covariates with lipid biomarkers in the EPIC–Heidelberg subcohort (*n* = 2739). Red depicts negative correlation, *blue* depicts positive correlation; the darker the shade the stronger the correlation. *apo* apolipoprotein, *HDL* high-density lipoprotein, *Lp(a)* lipoprotein a, *TC* total cholesterol, *TG* triglycerides

In the subcohort, HDL-C levels correlated strongly and positively with apo(a) (*r* = 0.85), and apoB-100 levels correlated strongly and positively with TC (*r* = 0.85) and less strongly but positively with TG (*r* = 0.42, Fig. 1). By contrast, HDL levels were negatively correlated with TG (*r* = –0.59). BMI and body weight correlated negatively with HDL-C (*r* = –0.35 and *r* = –0.33, respectively) and positively with TG (*r* = 0.34 and *r* = 0.31, respectively) in the subcohort. TC, TG, and apoB-100 levels were highest, and HDL and apo(a) levels lowest, among current smokers compared to never smokers (*P* for difference by smoking status < 0.01). Among postmenopausal women in the subcohort, current HRT users had lower Lp(a) levels compared to non-users (median 12 vs. 17 nmol/L, respectively; *P* = 0.008). Lp(a) did not correlate with any of the lipid biomarkers (*r* < 0.2) or further lifestyle factors (*P* > 0.05).

### Lipid biomarkers and risk of cancer incidence and mortality

**Fig. 2:**
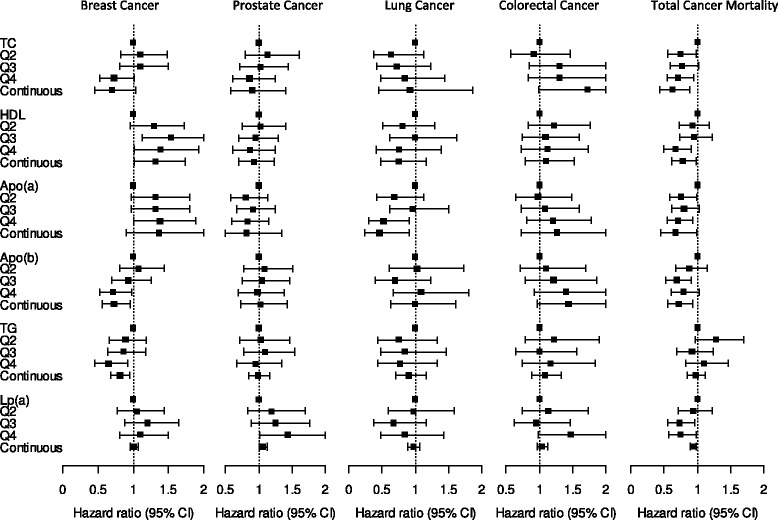
Associations of lipid biomarkers with site-specific cancer incidence and total cancer mortality, multivariable adjusted for sex, age at blood draw, baseline height, waist, BMI, lifetime alcohol consumption, red meat intake, fiber intake, smoking status, socioeconomic status, physical activity, diabetes, hypertension, and use of lipid-lowering drugs. *Q* quartile, *apo* apolipoprotein, *CI* confidence interval, *HDL* high-density lipoprotein, *Lp(a)* lipoprotein a, *TC* total cholesterol, *TG* triglycerides

Incident breast cancer risk was 1.45-fold higher among participants within the highest quartile of circulating apo(a) levels at baseline compared to those within the lowest quartile, whereas risks were 30% lower with high TG and high apoB-100 levels (highest vs. lowest quartile (Q4 vs. Q1, Q4 cut-offs 2.02, 1.90, and 1.24, respectively, crude models; Fig. 2, Additional file 2). TC and Lp(a) concentrations showed no significant associations with risk of breast cancer. Multivariable adjustments for body height, waist circumference, BMI, lifetime alcohol consumption, intake of red meat and fiber, smoking habits, socioeconomic status, physical activity, diabetes, hypertension, and use of lipid-lowering drugs had negligible effects on the associations of apoB-100 or TG with risk of breast cancer but attenuated the direct associations of high apo(a) levels with breast cancer risk to non-significance, resulting in significant direct associations of high HDL-C levels with risk of breast cancer (HR 1.39, Q4 cut-off 1.90). No single adjustment factor changed the risk estimates by more than 6%.

High Lp(a) levels were significantly associated with a 1.5 higher risk of incident prostate cancer (Q4 vs. Q1, crude HR 1.47, Q4 cut-off 42.45), while medium Lp(a) levels (Q2 and Q2) or other lipid markers were not associated with risk (Fig. 2, Additional file 3). Multivariable adjustments had negligible effects on this association.

Lung cancer risk was significantly lower with higher levels of apo(a) (Q4 vs. Q1, crude HR 0.44, Q4 cut-off 1.90) and higher levels of HDL-C (HR 0.46, Q4 cut-off 1.70), but significantly increased with high apoB-100 levels (HR 1.55, Q4 cut-off 1.30), whereas the other lipid biomarkers were not significantly associated with risk of lung cancer (Fig. 2, Additional file 4). High HDL-C and apoB-100 levels were no longer associated with lung cancer risk after multivariable adjustments, with smoking status being the strongest adjustment factor (change in HR by 26%), whereas the association of apo(a) with risk of lung cancer was slightly weakened but remained statistically significant. None of the lipid biomarkers showed significant associations with risk of colorectal cancer (Fig. 2, Additional file 5).

High TC, HDL-C, and apo(a) levels were all associated with a reduction in total cancer mortality (Q4 vs. Q1, crude models, HR_TC_ 0.71, HR_HDL-C_ 0.56, HR_apo(a)_ 0.64, Q4 cut-offs 6.60, 1.70, and 1.90, respectively; Table 2, Fig. 2). ApoB-100 and TG levels were not associated with cancer mortality, whereas high Lp(a) levels showed a borderline inverse association with total cancer mortality (HR 0.78, Q4 cut-off 45.40). Multivariable adjustments weakened the associations of HDL-C, slightly strengthened the associations of apo(a) and resulted in statistically significant associations of high Lp(a) levels with total cancer mortality (HR_Lp(a)_ 0.74), but did not materially alter the other results. The four most common causes of cancer death in our study were cancer of the lung (*n* = 138 cases out of 761, 18%), colorectum (*n* = 85, 11%), breast (*n* = 69, 9%), and pancreas (*n* = 60, 8%). High HDL-C levels were inversely associated with lung (for a doubling in concentration, sex-, age-, BMI-, and smoking-adjusted HR 0.56, 95% CI 0.32–0.98), high apoB-100 levels inversely associated with breast (HR 0.58, 95% CI 0.35–0.96), and high Lp(a) levels inversely with pancreas cancer mortality (HR 0.81, 95% CI 0.71–0.92), whereas colorectal cancer mortality showed no association with any of the lipids.

**Table 2:** Hazard ratios (HR) and 95% CI for associations of lipid biomarkers with total cancer mortality

Biomarker	quartile_1	quartile_2	quartile_3	quartile_4	pval_med	hr_con	pval_con
TC^a^	Ref	0.78 (0.60–1.00)	0.78 (0.61–1.01)	**0.77 (0.59–0.99)**	0.072	**0.67 (0.47–0.95)**	**0.025**
TC adjusted^b^	Ref	**0.74 (0.56–0.98)**	0.77 (0.59–1.02)	**0.71 (0.54–0.94)**	**0.036**	**0.62 (0.43–0.88)**	**0.009**
Median^c^	4.70/4.70/4.60	5.50/5.60/5.50	6.20/6.20/6.20	7.20/7.20/7.10			
No of cases	187	158	170	177			
HDL-C^a^	Ref	0.83 (0.66–1.03)	0.86 (0.69–1.08)	**0.56 (0.43–0.73)**	**<0.0001**	**0.67 (0.55–0.82)**	**0.0001**
HDL-C adjusted^b^	Ref	0.93 (0.73–1.18)	0.95 (0.74–1.22)	**0.67 (0.50–0.91)**	**0.021**	**0.78 (0.62–0.98)**	**0.037**
Median^c^	0.95/0.90/1.20	1.20/1.10/1.50	1.50/1.40/1.80	2.00/1.80/2.10			
No of cases	254	188	188	103			
apo(a)^a^	Ref	**0.77 (0.60–0.97)**	**0.75 (0.59–0.95)**	**0.64 (0.50–0.82)**	**0.0007**	**0.56 (0.39–0.80)**	**0.001**
apo(a) adjusted^b^	Ref	**0.76 (0.58–0.99)**	0.80 (0.62–1.03)	**0.71 (0.54–0.93)**	**0.029**	**0.67 (0.45–0.99)**	**0.044**
Median^c^	1.34/1.30/1.48	1.54/1.48/1.68	1.70/1.64/1.90	2.04/1.91/2.16			
No of cases	224	169	183	156			
apoB-100^a^	Ref	0.84 (0.66–1.08)	**0.73 (0.56–0.94)**	0.87 (0.68–1.12)	0.284	0.82 (0.64–1.05)	0.112
apoB-100 adjusted^b^	Ref	0.88 (0.68–1.15)	**0.69 (0.52–0.91)**	0.79 (0.60–1.03)	0.054	**0.72 (0.56–0.93)**	**0.011**
Median^c^	0.82/0.84/0.77	1.03/1.07/0.97	1.21/1.26/1.15	1.47/1.53/1.38			
No. of cases	173	178	160	204			
TG^a^	Ref	1.29 (1.00–1.67)	1.00 (0.77–1.29)	1.21 (0.94–1.56)	0.382	1.06 (0.95–1.19)	0.305
TG adjusted^b^	Ref	1.28 (0.97–1.70)	0.92 (0.69–1.23)	1.10 (0.82–1.46)	0.895	0.97 (0.85–1.11)	0.675
Median^c^	0.90/1.00/0.80	1.50/1.70/1.10	2.00/2.40/1.60	3.30/3.70/2.70			
No of cases	153	173	162	200			
Lp(a)^a^	Ref	0.98 (0.77–1.25)	**0.76 (0.59–0.98)**	0.78 (0.60–1.00)	0.080	**0.94 (0.90–0.99)**	**0.011**
Lp(a) adjusted^b^	Ref	0.94 (0.72–1.22)	**0.73 (0.56–0.96)**	**0.74 (0.57–0.98)**	0.076	**0.94 (0.89–0.98)**	**0.010**
Median^c^	3.50/3.30/3.85	8.50/8.10/9.40	20.25/19.40/21.75	129.60/121.60/134.70			
No of cases	189	187	160	157			

^a^Crude model adjusted for sex and age at blood draw

^b^Multivariable model further adjusted for baseline height, waist, BMI, lifetime alcohol consumption, red meat intake, fiber intake, smoking status, socioeconomic status, physical activity, diabetes, hypertension, and use of lipid-lowering drugs

^c^Medians of lipid parameters are of all/men/women within the cancer deaths

Entries in boldface are statistically significant

*apo* apolipoprotein, *CI* confidence interval, *HDL-C* high-density lipoprotein, *hr_con* continuous HR for a doubling in biomarker concentration, *Lp(a)* lipoprotein (a), *pval_con P*_trend_ continuously, *pval_med P*_trend_ over lipid biomarker levels based on the median of each quartile, *TC* total cholesterol, *TG* triglycerides

### Lipid biomarkers and risk of CVD incidence and mortality

**Fig. 3:**
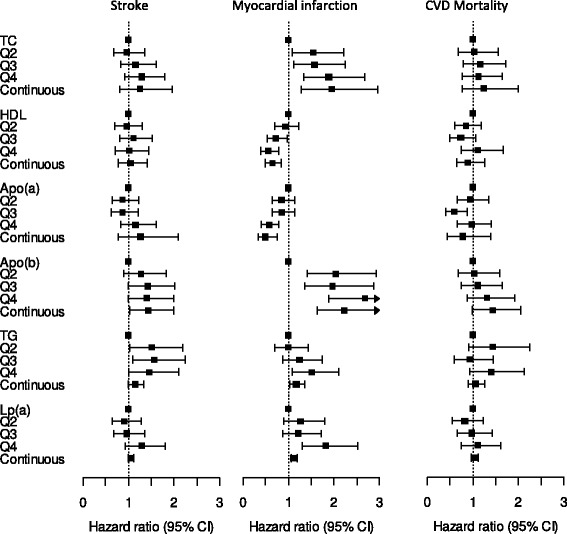
Associations of lipid biomarkers with incident myocardial infarction, incident stroke, and cardiovascular mortality, multivariable adjusted for sex, age at blood draw, baseline height, waist, BMI, lifetime alcohol consumption, *red* meat intake, fiber intake, smoking status, socioeconomic status, physical activity, diabetes, hypertension, and use of lipid-lowering drugs. *apo* apolipoprotein, *CI* confidence interval, *HDL* high-density lipoprotein, *Lp(a)* lipoprotein (a), *Q* quartile, *TC* total cholesterol, *TG* triglycerides

Only higher TG levels were significantly associated with risk of stroke (Fig. 3 and Additional file 6), whereas all of the lipid biomarkers were significantly associated with MI in both crude and multivariable adjusted models, with TC, apoB-100, TG, and Lp(a) showing positive and HDL-C and apo(a) showing inverse associations, confirming findings from previous studies (Fig. 3 and Additional file 7). Multivariable adjustments slightly strengthened the association of Lp(a) with risk of MI, but attenuated those of the other markers with risk of MI. Waist circumference was the strongest adjustment factor for the associations of TC, HDL and TG with risk of MI (change in HRs by 21% to 28%). Further multivariable adjusted analyses by stroke subtypes revealed significant direct associations of high Lp(a) levels with risk of ischemic but not hemorrhagic stroke (Q4 vs. Q1, HR 1.60, 95% CI 1.04–2.46), whereas other biomarkers were not associated with either stroke subtype (data not shown).

High HDL-C and apo(a) levels were inversely associated with CVD mortality whereas apoB-100 and TG were positively associated with CVD mortality (Fig. 3 and Additional file 8). However, none of the hazard ratios for CVD mortality remained statistically significant after multivariable adjustments, whereby waist circumference and BMI had the largest effects on risks (change in HRs by 24% to 37%).

### Subgroup analyses for cancer mortality and incidence

We observed significant heterogeneity by menopausal status at baseline, but not by age 55 at diagnosis, for the associations of HDL-C and apo(a) with breast cancer risk, with a significant increase in risk among premenopausal but not postmenopausal women (age- and BMI-adjusted, for a doubling in concentration, HDL-C: HR_pre_ 1.64, 95% CI 1.17–2.30 and HR_post_ 0.83, 0.51–1.35; *P* = 0.024; and apo(a): HR_pre_ 2.13, 1.33–3.40) and HR_post_ 0.83, 0.46–1.51; *P* = 0.008). Within postmenopausal women at baseline, significant heterogeneity by HRT use was observed for the association of Lp(a) levels with breast cancer risk (*P* = 0.002), with a significant Lp(a)-associated increase in breast cancer risk among HRT users compared to a significant inverse association among non-users (HR_yes_ 1.09, 1.01–1.81; HR_no_ 0.93, 0.87–1.00). Regarding lung cancer, the inverse associations of high HDL-C and apo(a) levels with risk of lung cancer were confined to current smokers due to significant heterogeneity by smoking status (age-, sex-, and BMI-adjusted, HDL-C: *P* < 0.001, HR_current_ 0.38, 0.22–0.66; and apo(a): *P* < 0.001, HR_current_ 0.20, 0.09–0.48). The associations of TC and HDL-C levels with cancer mortality were also modified by smoking status, such that high TC and HDL-C levels were associated with a significantly reduced cancer mortality among never and former smokers, but not among current smokers after adjustments for sex, age, and BMI (TC: *P* = 0.028, HR_never_ 0.50, 0.28–0.89; HR_former_ 0.38, 0.20–0.70; HR_current_ 1.21, 0.64–2.32; and HDL-C: *P* = 0.029, HR_never_ 0.68, 0.45–0.99; HR_former_ 0.51, 0.33–0.80; HR_current_ 1.19, 0.76–1.86). We did not observe further heterogeneity by HRT use or smoking status for any of the other diseases or mortality endpoints, and we also observed no heterogeneity by sex, current alcohol consumption at baseline (≤ 6 vs. > 6 g/day) or obesity (e.g., below or above a BMI of 25 kg/m^2^) for the associations of lipid biomarkers with risk of site-specific cancer or cancer mortality.

Excluding participants with less than 2 years of follow-up between recruitment and cancer diagnosis did not materially alter any of the results, whereas the extension to 5 years slightly attenuated the associations of TG with breast cancer, Lp(a) with prostate cancer, and apo(a) with total cancer mortality. Exclusion of participants with prevalent liver or kidney insufficiency also had negligible effects on risk estimates (data not shown). Excluding women using HRT at baseline resulted in a reduction of breast cancer cases of up to 65% and diminished the associations of HDL-C, apo(a), and TG to non-significance but resulted in significant associations of TC with risk of breast cancer in similar strength to the associations within the full study population (Q4 vs. Q1, HR_HDL_ 1.24, 0.85–1.80; HR_apo(a)_ 1.19, 0.83–1.71; HR_TG_ 0.73, 0.50–1.07; HR_TC_ 0.65, 0.43–0.99). Excluding participants using lipid-lowering drugs at baseline resulted in significant risk estimates for apoB-100 with stroke (Q4 vs. Q1, HR 1.53, 1.07–2.18) and slightly stronger estimates for TC and apoB-100 with MI (HR_TC_ 2.03, 1.43–2.87; HR_apoB_ 2.93, 2.05–4.20), whereas other results were not different to the full study population.

### Applying clinical cut-points for lipid biomarkers

Using the clinical cut-points for TC ≥ 6.18, HDL ≤ 1.03, apo(a) ≤ 1.05, apoB ≥ 1.56, TG ≥ 2.26, and Lp(a) ≥ 120, high TG and Lp(a) levels were associated with risk of stroke and high TC, TG, apoB, and Lp(a) levels with risk of MI after multivariable adjustment in comparable strength to quartile analyses mirroring these cut-points (Additional file 9).

## Discussion

In this prospective study of middle-aged German men and women with more than 1600 cases of cancer and 1000 of CVD, and with almost 800 deaths caused by cancer and 400 by CVD, we found that all lipid biomarkers were associated with risk of MI, whereby TC, apoB-100, TG, and Lp(a) were positively and HLD-C and apo(a) inversely associated with risk, as expected. Further, high levels of TG were associated with an increased risk of stroke. For cancer, by contrast, we observed that high levels of circulating apoB-100 and TG were inversely and high HDL-C levels were positively associated with breast cancer risk. Further, higher levels of Lp(a) were associated with an increase in prostate cancer risk and high levels of apo(a) were associated with a decrease in lung cancer risk, whereas none of the lipids were associated with risk of colorectal cancer. With regard to mortality, high TC, HDL-C, apo(a), and Lp(a) levels were associated with a reduction in total cancer mortality, whereas none of the lipids were associated with risk of CVD mortality. Thus, the observed cancer risk pattern clearly differs from the CVD risk pattern.

### Plausibility

A blood lipid profile characterized by high TC, LDL-C, TG, apoB-100, and Lp(a) and by low HDL-C and apo(a) is a well-established risk profile for CVD events, in particular for MI and other coronary heart diseases, and our study fully confirms these associations. A direct translation of this CVD risk pattern to a similar cancer risk pattern appears to be difficult, however, as results from prospective studies are not fully consistent and precise biological mechanisms linking lipid biomarkers to cancer development or cancer death are largely unknown. Several biological mechanisms have been proposed to account for an association between altered blood cholesterol levels and cancer incidence or mortality, including immune system disturbances, altered protein function, low circulating levels of fat-soluble anti-oxidants, increased tumor angiogenesis, reduced tumor apoptosis and increased tumor cell proliferation [21, 22]. Experimental and animal studies have shown that both HDL-C and apo(a), the predominant protein in plasma HDL-C, may protect against tumor development via manifold mechanisms such as influencing signaling pathways by modulating cholesterol content in cell membranes [39]. Experimental studies have also shown that HDL-C presents antioxidant and anti-inflammatory properties and plays a role in the inhibition of the LDL oxidation cascade [40, 41]. A further possible mechanism involving apo(a) includes the inhibition of cell proliferation and cell-cycle progression [41]. Hypertriglyceridemia, by contrast, has been speculated to be associated with the development of oxidative stress and the formation of reactive oxygen species, which play an important role in normal cell proliferation and are increased in cancer cells [42].

### Lipid biomarkers and risk of incidence cancer

Previous prospective investigations showed inverse or null associations for most blood lipids with risks of breast, prostate, colorectal, or lung cancer. For breast cancer, we observed a positive association with HDL-C levels above 1.90, which contradicts with the latest meta-analysis in 2015 and prospective cohort studies that have been conducted thereafter [9, 11, 12, 16]. In one further prospective nested-case control study, HDL was positively associated with breast cancer risk in a similar magnitude to our observed hazard ratio of 1.45 comparing the highest with the lowest quartile [43]. Interestingly, our observed positive association of HDL-C with breast cancer risk is confined to premenopausal women at baseline, as previously shown [44], mirroring the negative correlation of BMI with HDL-C levels (*r* = –0.35) and the inverse association of excess body weight with premenopausal breast cancer risk, as observed in EPIC but also other prospective studies [45, 46]. Opposite to HDL-C, TG (≥ 1.90) and apoB-100 levels (≥ 1.24) were inversely associated with breast cancer risk in our study, whereas the current literature only reports null associations thus far [9, 12, 14]. HRT use seemed to be an important effect modifier for the associations of Lp(a) with breast cancer risk. Investigations on HRT-induced changes in lipid parameters among postmenopausal women showed a decrease in Lp(a) levels of almost 30% after 6 or more months using HRT [47, 48]. Among the women in our healthy subcohort, Lp(a) levels were also lower in women using HRT compared to non-users. In our data, an increase in Lp(a) levels was related to higher risk of breast cancer among postmenopausal women using HRT, even though HRT users had lower overall Lp(a) levels compared to non-users.

Evidence for the associations of lipid biomarkers with risk of lung cancer is limited to two prospective studies, as far as we know [9, 11]. Similar to these two studies, but with a much stronger hazard ratio, we observed a significant risk reduction of lung cancer with apo(a) levels above 1.90. However, this association was confined to current smokers and we observed a significant effect modification by smoking status.

### Lipid biomarkers and mortality

A limited number of epidemiological studies have investigated the associations of total cholesterol with overall cancer mortality and, as in our study, they all showed that low total cholesterol levels are associated with greater cancer mortality [23, 24, 27]. Prospective studies on the association of HDL-C and apo(a) with cancer mortality are very few. One Korean study showed a 50% reduction in cancer mortality with higher apo(a) levels, but no association of HDL-C with cancer mortality [26], similar to findings from an older cohort in US Americans in the 1970s [27]. Our data also showed a significantly lower cancer mortality with both high HDL-C and high apo(a) levels, which is in line with a recent investigation in the Women’s Health study [9]. The association of Lp(a) with cancer mortality has been investigated in only one other prospective study so far [28], showing an inverse association, in line with our findings. Our results on cancer mortality are not modified by lifestyle habits, with the only exception of a possible effect modification by smoking status in the inverse association of TC and HDL-C with cancer mortality.

Despite the abovementioned possible biological mechanisms, a common metabolic profile characteristic for cancer similar to the one for CVD does not clearly emerge from our and previous prospective investigations. The opposite directions of the associations between HDL-C, apoB-100, and TG with breast cancer and the inverse associations between most of the lipid parameters and cancer death point towards distinct differences by endpoint. Indeed, cell line and animal studies have shown that intracellular cholesterol homeostasis varies among different cancer types, and therefore cholesterol could play differing roles depending on cancer type [49].

Mendelian randomization studies, where genetic variants as proxies for altered levels of an exposure are used to assess the causal relationship with the outcome, might shed light on the complex and still unknown associations of lipids with risk of cancer and cancer mortality. However, these Mendelian randomization studies have scarcely been conducted so far and are limited to overall cancer (two studies) or prostate cancer (one study) and did not show any causal relationships of circulating lipids, instrumented by genetic risk scores, with risk [50–52].

### Strengths and limitations

A potential limitation of our study could be reverse causation bias. However, exclusion of cases with less than 2 years of follow-up did not alter any of the observed associations. Further, measuring lipid biomarkers at one point in time might not reflect fluctuations of levels over time. Lp(a) concentrations are relatively refractory to changes in lifestyle or drug use [53] and, thus, a single-time measurement of Lp(a) reasonably well reflects average levels over time within one person. All other lipid biomarkers have also been shown to be quite stable over time periods of multiple years [54]. Strengths of our study include its prospective design, the large number of cancer cases and deaths attributable to cancer, well-standardized measurements of lipid markers, and comprehensive information on many potential confounding factors that we could account for, including lifestyle, medical history, and medication use. In addition, the strong positive correlations of HDL-C with apo(a) and of TC with apoB-100 as well as the strong inverse correlations of TG with HDL-C in our study are as biologically expected and strengthen our results. Further, our study showed that high levels of TC, apoB-100, TG, and Lp(a) were associated with an increased risk of MI, whereas high levels of HDL-C and apo(a) were associated with a lower risk. These observations are fully in line with the literature [55] and, thus, lend credence to our findings on the relationships of lipids with risk of the most common cancer entities.

## Conclusions

In conclusion, in this large prospective case-cohort study we found that high apo(a) levels were associated, as expected from the literature, with a decreased lung cancer risk. In contrast to previous investigations, high HDL-C levels were associated with an increased breast cancer risk and high TG and apoB-100 levels were inversely associated with breast cancer risk. Finally, TC, HDL-C, apo(a), and Lp(a) levels were associated with a reduction in cancer mortality. Thus, the observed cancer risk pattern clearly differs from the CVD risk pattern. More prospective studies with a detailed assessment of lifestyle and medical variables at baseline are needed to further evaluate (1) the specific role of HDL-C and Lp(a) in breast cancer development and their interaction with exogenous sex hormones, (2) the role of lipid biomarkers in lung cancer development, (3) the role of Lp(a) in any cancer development, (4) the effect of all lipid biomarkers on cancer mortality, and (5) the possible effect modification by HRT use and smoking status. Complementary, Mendelian randomization studies might potentially add valuable insights of any causal relationship.

## Additional files

Additional file 1: Hazard ratios and 95% confidence intervals for associations of low-density lipoprotein cholesterol with incident cancers, cancer mortality, incident cardiovascular disease (CVD), and CVD mortality. (DOC 68 kb)

Additional file 2: Hazard ratios and 95% confidence intervals for associations of lipid biomarkers with incident breast cancer. (DOC 64 kb)

Additional file 3: Hazard ratios and 95% confidence intervals for associations of lipid biomarkers with incident prostate cancer. (DOC 63 kb)

Additional file 4: Hazard ratios and 95% confidence intervals for associations of lipid biomarkers with incident lung cancer. (DOC 64 kb)

Additional file 5: Hazard ratios and 95% confidence intervals for associations of lipid biomarkers with incident colorectal cancer. (DOC 64 kb)

Additional file 6: Hazard ratios and 95% confidence intervals for associations of lipid biomarkers with incident stroke. (DOC 64 kb)

Additional file 7: Hazard ratios and 95% confidence intervals for associations of lipid biomarkers with incident myocardial infarction. (DOC 64 kb)

Additional file 8: Hazard ratios and 95% confidence intervals for associations of lipid biomarkers with cardiovascular disease mortality. (DOC 64 kb)

Additional file 9: Hazard ratios and 95% confidence intervals for associations of lipid biomarkers with incident cancers, cancer mortality, incident cardiovascular disease (CVD) and CVD mortality, using clinical cut-offs. (DOC 66 kb)

## Abbreviations

apo(a): apolipoprotein (a)
apoB-100: apolipoprotein B-100
CI: Confidence interval
CVD: Cardiovascular diseases
EPIC: European prospective investigation into cancer and nutrition
HDL: High-density lipoprotein
HR: Hazard ratio
HRT: Hormone replacement therapy
ICD: International classification of diseases
LDL: Low-density lipoprotein
Lp(a): Lipoprotein(a)
MI: Myocardial infarction
TC: Total cholesterol
TG: Triglycerides
