# Supplementary material for: Blood lipids and lipoproteins in relation to incidence and mortality risks for CVD and cancer in the prospective EPIC–Heidelberg cohort
Source: BMC Med. 2017 Dec 19;15:218. doi: 10.1186/s12916-017-0976-4 (PMC5735858; doi:10.1186/s12916-017-0976-4)
Supplement: Supplementary file 1 — Hazard ratios and 95% confidence intervals for associations of low-density lipoprotein cholesterol with incident cancers, cancer mortality, incident cardiovascular disease (CVD), and CVD mortality. (DOC 68 kb) [file 12916_2017_976_MOESM1_ESM.doc]

| **Additional file 1** Hazard ratios (HR) and 95% CI for associations of **LDL-C with incident cancers, cancer mortality, incident CVD and CVD mortality** | | | | | | | |
| --- | --- | --- | --- | --- | --- | --- | --- |
| Endpoint | quartile_1 | quartile_2 | quartile_3 | quartile_4 | pval_med | hr_con | pval_con |
|  |  |  |  |  |  |  |  |
| **Breast cancer** | 2.71 | 3.60 | 4.14 | 4.97 |  |  |  |
| Crude* | Ref | 1.06 (0.80,1.40) | 0.89 (0.67,1.18) | **0.66 (0.48,0.89)** | **0.002** | **0.71 (0.56,0.91)** | **0.007** |
| Adjusted** | Ref | 1.09 (0.81,1.46) | 0.97 (0.71,1.31) | **0.71 (0.52,0.99)** | **0.023** | 0.77 (0.59,1.00) | 0.050 |
|  |  |  |  |  |  |  |  |
| **Prostate cancer** | 3.15 | 3.84 | 4.42 | 5.24 |  |  |  |
| Crude* | Ref | 1.08 (0.78,1.49) | 1.01 (0.73,1.41) | 0.91 (0.65,1.27) | 0.454 | 1.00 (0.75,1.33) | 0.979 |
| Adjusted** | Ref | 1.11 (0.79,1.57) | 1.00 (0.70,1.43) | 0.88 (0.61,1.27) | 0.357 | 0.98 (0.72,1.33) | 0.878 |
|  |  |  |  |  |  |  |  |
| **Lung cancer** | 3.00 | 3.67 | 4.24 | 5.38 |  |  |  |
| Crude* | Ref | 0.97 (0.60,1.58) | 0.96 (0.59,1.57) | 1.58 (1.00,2.49) | **0.034** | 1.50 (0.94,2.39) | 0.091 |
| Adjusted** | Ref | 1.12 (0.63,1.99) | 0.75 (0.41,1.38) | 1.17 (0.67,2.02) | 0.687 | 1.07 (0.65,1.76) | 0.783 |
|  |  |  |  |  |  |  |  |
| **Colorectal cancer** | 3.03 | 3.80 | 4.36 | 5.50 |  |  |  |
| Crude* | Ref | 0.95 (0.61,1.48) | 1.08 (0.70,1.65) | 1.24 (0.82,1.89) | 0.212 | 1.47 (0.99,2.16) | 0.053 |
| Adjusted** | Ref | 0.96 (0.61,1.51) | 1.09 (0.70,1.71) | 1.20 (0.77,1.88) | 0.305 | 1.42 (0.95,2.13) | 0.086 |
|  |  |  |  |  |  |  |  |
| **Cancer death** | 2.90 | 3.70 | 4.40 | 5.32 |  |  |  |
| Crude* | Ref | 0.87 (0.68,1.13) | 0.77 (0.59,1.00) | 0.81 (0.62,1.04) | 0.091 | 0.84 (0.66,1.07) | 0.157 |
| Adjusted** | Ref | 0.92 (0.70,1.21) | **0.75 (0.57,0.99)** | **0.74 (0.56,0.97)** | **0.016** | **0.77 (0.60,0.98)** | **0.037** |
|  |  |  |  |  |  |  |  |
| **Stroke** | 2.88 | 3.78 | 4.38 | 5.38 |  |  |  |
| Crude | Ref | 1.00 (0.71,1.39) | 1.18 (0.86,1.63) | 1.26 (0.92,1.74) | 0.093 | 1.24 (0.92,1.68) | 0.164 |
| Adjusted | Ref | 1.01 (0.71,1.42) | 1.16 (0.83,1.63) | 1.08 (0.77,1.53) | 0.514 | 1.08 (0.80,1.47) | 0.616 |
|  |  |  |  |  |  |  |  |
| **MI** | 3.04 | 3.79 | 4.40 | 5.36 |  |  |  |
| Crude* | Ref | **1.55 (1.09,2.20)** | **2.09 (1.49,2.94)** | **2.54 (1.82,3.56)** | **<0.0001** | **2.36 (1.76,3.17)** | **<0.0001** |
| Adjusted** | Ref | 1.40 (0.95,2.05) | **1.98 (1.38,2.85)** | **2.05 (1.43,2.95)** | **<0.0001** | **1.94 (1.43,2.64)** | **<0.0001** |
|  |  |  |  |  |  |  |  |
| **CVD death** | 2.84 | 3.74 | 4.40 | 5.33 |  |  |  |
| Crude* | Ref | 1.10 (0.75,1.61) | 1.27 (0.88,1.84) | 1.41 (0.97,2.04) | **0.043** | **1.45 (1.03,2.03)** | **0.032** |
| Adjusted** | Ref | 1.09 (0.71,1.67) | 1.21 (0.81,1.80) | 1.13 (0.76,1.68) | 0.509 | 1.15 (0.82,1.61) | 0.414 |
| *Crude model adjusted for sex and age at blood draw. **Multivariable model further adjusted for baseline height, waist, BMI, lifetime alcohol consumption, red meat intake, fibre intake, smoking status, socioeconomic status, physical activity, diabetes, hypertension, use of lipid lowering drugs and for breast cancer further for oral contraceptive use, hormone replacement therapy, menopausal status and full-term pregnancies. CI = confidence interval, LDL-C = low-density lipoprotein. MI = myocardial infarction, CVD = cardiovascular diseases. The Friedewald formula (LDL = TC – HDL - TG/5) was applied to calculate LDL-C values. | | | | | | | |
