# Supplementary material for: Blood lipids and lipoproteins in relation to incidence and mortality risks for CVD and cancer in the prospective EPIC–Heidelberg cohort
Source: BMC Med. 2017 Dec 19;15:218. doi: 10.1186/s12916-017-0976-4 (PMC5735858; doi:10.1186/s12916-017-0976-4)
Supplement: Supplementary file 7 — Hazard ratios and 95% confidence intervals for associations of lipid biomarkers with incident myocardial infarction. (DOC 64 kb) [file 12916_2017_976_MOESM7_ESM.doc]

| **Additional file 7** Hazard ratios (HR) and 95% CI for associations of lipid biomarkers with **incident myocardial infarction** | | | | | | | |
| --- | --- | --- | --- | --- | --- | --- | --- |
| Biomarker | quartile_1 | quartile_2 | quartile_3 | quartile_4 | pval_med | hr_con | pval_con |
|  |  |  |  |  |  |  |  |
| TC* | Ref | **1.62 (1.17,2.25)** | **1.68 (1.21,2.32)** | **2.11 (1.53,2.91)** | **<0.0001** | **2.43 (1.63,3.63)** | **<0.0001** |
| TC adjusted** | Ref | 1.54 (1.07,2.21) | **1.57 (1.11,2.24)** | **1.88 (1.33,2.66)** | **0.001** | **1.94 (1.28,2.96)** | **0.002** |
| Median*** | 4.80/4.90/4.60 | 5.60/5.60/5.45 | 6.20/6.20/6.25 | 7.20/7.20/7.20 |  |  |  |
| No of cases | 75 | 116 | 132 | 165 |  |  |  |
|  |  |  |  |  |  |  |  |
| HDL-C* | Ref | **0.73 (0.56,0.93)** | **0.56 (0.43,0.73)** | **0.35 (0.26,0.49)** | **<0.0001** | **0.44 (0.36,0.55)** | **<0.0001** |
| HDL-C adjusted** | Ref | 0.93 (0.70,1.22) | **0.72 (0.53,0.96)** | **0.55 (0.38,0.78)** | **<0.001** | **0.65 (0.50,0.84)** | **<0.001** |
| Median*** | 0.90/0.90/1.10 | 1.20/1.10/1.50 | 1.40/1.40/1.80 | 1.90/1.80/2.30 |  |  |  |
| No of cases | 224 | 141 | 114 | 58 |  |  |  |
|  |  |  |  |  |  |  |  |
| apo(a)* | Ref | **0.76 (0.59,0.99)** | **0.71 (0.55,0.93)** | **0.43 (0.32,0.57)** | **<0.0001** | **0.30 (0.21,0.45)** | **<0.0001** |
| apo(a) adjusted** | Ref | 0.84 (0.63,1.13) | 0.85 (0.64,1.14) | **0.57 (0.41,0.79)** | **0.002** | **0.49 (0.32,0.75)** | **0.001** |
| Median*** | 1.31/1.28/1.46 | 1.50/1.48/1.67 | 1.65/1.63/1.89 | 1.90/1.82/2.18 |  |  |  |
| No of cases | 180 | 136 | 138 | 83 |  |  |  |
|  |  |  |  |  |  |  |  |
| apoB-100* | Ref | **1.97 (1.39,2.78)** | **2.20 (1.56,3.10)** | **3.16 (2.27,4.41)** | **<0.0001** | **2.91 (2.17,3.92)** | **<0.0001** |
| apoB-100 adjusted** | Ref | **2.03 (1.40,2.93)** | **1.97 (1.36,2.86)** | **2.68 (1.88,3.84)** | **<0.0001** | **2.22 (1.63,3.03)** | **<0.0001** |
| Median*** | 0.85/0.89/0.80 | 1.06/1.08/1.01 | 1.26/1.28/1.17 | 1.52/1.54/1.44 |  |  |  |
| No of cases | 57 | 128 | 142 | 207 |  |  |  |
|  |  |  |  |  |  |  |  |
| TG* | Ref | 1.21 (0.87,1.67) | **1.50 (1.10,2.04)** | **2.13 (1.58,2.86)** | **<0.0001** | **1.42 (1.26,1.61)** | **<0.0001** |
| TG adjusted** | Ref | 0.99 (0.69,1.43) | 1.24 (0.88,1.74) | **1.51 (1.08,2.10)** | **0.006** | **1.17 (1.02,1.35)** | **0.029** |
| Median*** | 1.10/1.20/0.80 | 1.60/1.70/1.20 | 2.20/2.30/1.60 | 3.90/4.05/2.50 |  |  |  |
| No of cases | 90 | 89 | 126 | 181 |  |  |  |
|  |  |  |  |  |  |  |  |
| Lp(a)* | Ref | 1.34 (0.98,1.84) | 1.22 (0.88,1.67) | **1.77 (1.31,2.39)** | **<0.001** | **1.10 (1.05,1.16)** | **<0.001** |
| Lp(a) adjusted** | Ref | 1.26 (0.89,1.79) | 1.22 (0.87,1.72) | **1.81 (1.30,2.52)** | **<0.001** | **1.11 (1.05,1.18)** | **<0.001** |
| Median*** | 3.35/3.30/3.70 | 8.85/8.80/9.70 | 20.90/21.10/19.90 | 138.00/148.90/ 120.90 |  |  |  |
| No of cases | 88 | 118 | 115 | 161 |  |  |  |
| *Crude model adjusted for sex and age at blood draw. **Multivariable model further adjusted for baseline height, waist, BMI, lifetime alcohol consumption, red meat intake, fibre intake, smoking status, socioeconomic status, physical activity, diabetes, hypertension and use of lipid lowering drugs. ***Medians of lipid parameters are of all / men / women within the myocardial infarction cases. CI = confidence interval, pval_med = p-trend over lipid biomarker levels based on the median of each quartile, hr_con = continuous HR for a doubling in biomarker concentration, pval_con = p-trend continuously, No = number, TC = total cholesterol, HDL-C = high-density lipoprotein, apo = apolipoprotein, TG = triglycerides, Lp(a) = lipoprotein (a) | | | | | | | |
